# Differential impact of hormone receptor status on survival and recurrence for HER2 receptor-positive breast cancers treated with Trastuzumab

**DOI:** 10.1007/s10549-017-4225-5

**Published:** 2017-04-04

**Authors:** Andrew McGuire, Olga Kalinina, Emma Holian, Catherine Curran, Carmel A. Malone, Ray McLaughlin, Aoife Lowery, James A. L. Brown, Michael J. Kerin

**Affiliations:** 1 Discipline of Surgery, Lambe Institute for Translational Research, School of Medicine, National University of Ireland Galway, Galway, Ireland; 2 School of Mathematics, Statistics and Applied Mathematics, National University of Ireland Galway, Galway, Ireland

**Keywords:** HER2, Trastuzumab, Luminal B, Luminal B HER2, HER2, HER2+(ER−), Breast cancer, Survival, Metastasis, Distant

## Abstract

**Introduction:**

Hormone receptor status has major implications for treatment and survival of breast cancer. Yet the impact of hormone receptor status on outcome after Trastuzumab has received little attention. The objective here was to explore any differential effects of Trastuzumab treatment (Trast +ve) on Luminal B HER2 or HER2+(ER−) breast cancer subtypes.

**Methods:**

A cohort of 469 HER2 receptor-positive breast cancers was categorised by molecular subtype and Trastuzumab treatment. Effects of Trastuzumab treatment on survival, locoregional recurrence and distant metastasis were investigated by subtype, using univariate and multivariate analysis.

**Results:**

Trast +ve Luminal B HER2 patients had significant improvements in 5-year DFS (*p* < 0.001) and OS (*p* < 0.001), while Trast +ve HER2+(ER−) patients had significant improvements in 5-year DFS (*p* = 0.012) alone. Only Trast +ve Luminal B HER2 cancers displayed a significant reduction in LRR rates (*p* < 0.001). A significant reduction in distant metastasis rates was seen in Trast +ve Luminal B HER2 (*p* < 0.001) and HER2+(ER−) (*p* = 0.009) cancers. Interestingly, bone metastasis rates in Trast +ve Luminal B HER2 cancers demonstrated the greatest reduction (36.2–6.7%). Multivariate analysis of Trast +ve patients found no difference in distant metastasis rates (*p* = 0.96) between subtypes. Significantly, lower LRR rates were seen in Trast +ve Luminal B HER2 cancers, compared to Trast +ve HER2+(ER−) (*p* = 0.018).

**Conclusion:**

An enhanced response to Trastuzumab was seen in Luminal B HER2 cancers. We highlight how Trastuzumab treatment changed the natural history of the HER2 receptor-positive breast cancer, demonstrating improved efficacy in changing the outcome of hormone receptor-positive patients.

**Electronic supplementary material:**

The online version of this article (doi:10.1007/s10549-017-4225-5) contains supplementary material, which is available to authorized users.

## Introduction

Advances in molecular profiling have allowed breast cancer to be categorised into clinically relevant molecular subtypes. [1–3]. In approximately 20–30% of breast cancers, the HER2 receptor is over expressed [4, 5], resulting in increased cell signalling, uncontrolled cellular proliferation and poor clinical prognosis. Half of these are hormone receptor-positive Luminal B HER2 and half are hormone receptor-negative HER2+(ER−). Differences in survival and outcome occur between the two HER2 receptor-positive subtypes [6, 7]. However, some studies have shown no significant difference when assessing long-term survival between the two subtypes [8–10]. Clinically, the two subtypes present with distinct patterns of recurrence. In Luminal B HER2 subtype bone is the most common distant metastasis site, similar to Luminal A breast cancers [8, 11]. HER2+(ER−) cancers have the highest rates of locoregional recurrence (LLR) overall and tend to initially metastasise to visceral organs, such as the lung [8, 12].

Trastuzumab is a monoclonal antibody that binds to the HER2 receptor and interferes with the HER2-mediated signalling cascade, preventing proliferation and eventually leading to cell death [13]. Trastuzumab was originally used to treat metastatic breast cancers and was shown to significantly improve median survival from 20.3 to 25.1 months [14]. Multiple studies have shown that Trastuzumab used in the adjuvant setting reduces recurrences and can increase survival in HER2-positive patients by up to 33–39% [15–18]. More recently, several studies have shown that when used in the neo-adjuvant setting Trastuzumab significantly increases pathological complete response (pCR) rates [19, 20].

While improvements in survival in HER2 receptor-positive cancers have been demonstrated in multiple studies, few studies have examined if Trastuzumab treatment has a varied response between Luminal B HER2 and HER2+(ER−) breast cancers. The aim of this study was to assess the impact of Trastuzumab therapy on the survival or outcome on the two HER2 receptor-positive breast cancer subtypes, before and after the introduction of Trastuzumab as part of the adjuvant treatment regime.

## Methods

### Patient cohort

This study group consists of all patients with HER2 receptor-positive breast cancers treated at a tertiary referral unit entered into a prospectively maintained database from 1991 to 2014. Only patients with a definitive HER2 receptor-positive subtype were included. All clinical pathological details and treatment regimes were analysed. Hormone receptor-positive patients received hormone therapy, as per standard clinical treatment protocols at the time of diagnosis. Clinically, testing for HER2 receptor status began at our centre in 1999. In order to find HER2 receptor-positive patients not treated with Trastuzumab, we used a cohort of patients identified using retrospective testing of pathology samples. A cohort of HER2 receptor-positive patients who received no Trastuzumab treatment was provided by retrospective testing of HER2 receptor status on patients included on a prospectively collected tissue microarray (samples from 1994 to 2001). Historically in our program, Trastuzumab therapy was introduced as adjuvant therapy in 2006, prior to this it was available only to patients recruited on clinical trials. Patients were categorised as received adjuvant/neo-adjuvant Trastuzumab (Trast +ve) or no Trastuzumab treatment (Trast −ve).

### Subtypes definitions

Breast cancer subtypes were defined using ER, PR and HER2 receptor status. Luminal B HER2 was defined as (ER and/or PR +ve, HER2 +ve) and HER2+(ER−) as (ER and PR –ve, HER2 +ve) according to standard clinical pathological guidelines. The ER and PR receptor status were determined independently by clinical pathologists using immunohistochemistry as per ASCO guidelines (ALLRED score >2 or more than 1% stain positive). The HER2 receptor status was identified by Herceptest, as part of the routine clinical evaluation, with a score of 3+ considered positive. Any +2 inconclusive results were confirmed using a FISH testing as per ASCO guidelines, with a HER2/CEP17 ratio greater than two considered amplified.

### Survival

Overall survival (OS), disease free survival (DFS), and patterns of recurrence were determined. The 5-year DFS & OS were determined and only patients who had completed 5 years of follow-up were included in the analysis.

### Recurrence

Breast cancer recurrence was defined as a return of cancer after treatment and after a disease free period. Only stage I–III breast cancers were included for this section of the analysis. Recurrence was divided into LRR and distant metastasis. LRR is defined as recurrence at the same site of the primary cancer or the regional lymph nodes, while distant metastasis is recurrence at a distant site from the primary cancer.

### Statistics

Statistical analysis was performed using R statistical software version 3.2.3. A *p* value of less than 0.05 was considered statistically significant. The Kaplan–Meier method was used to determine survival distributions. The log rank was used to determine any statistically significant differences in survival between the indicated groups. Cox regression was used for multivariate analysis, with logistic regression used to analyse categorical data.

### Ethics, consent and permissions

This study was conducted in accordance with the granted National University of Ireland Galway and University College Hospital Galway ethical approval. Informed consent was obtained from all patients. All patients had histologically confirmed breast cancer and all relevant clinicopathological and demographic data were obtained from a prospective breast cancer database.

## Results

### Cohort description

The study consisted of 468 HER2 receptor-positive patients eligible for this study, treated in our institute between 1991 and 2014. From these, 287 (61%) were found to be Luminal B HER2, with the remaining 181 (39%) patients HER2+(ER−). The median age of patients was 63 and the median follow-up was 49 months. The majority of the overall cohort was recruited after Trastuzumab received approval for adjuvant treatment in 2006 (Table 1). For the purpose of this analysis, patients were categorised as either Trast +ve (received adjuvant/neo-adjuvant Trastuzumab) or Trast −ve (no Trastuzumab treatment).

**Table 1:** Demographics

	Luminal B HER2 (*n* = 287) *N* (%)	HER2+(ER−) (*n* = 181) *N* (%)	*p* Value
Age: mean, Years±SD	64.62 ± SD	62.83 ± SD	0.160
Age category: *N* (%)	14.81	12.33	0.602
0–50	53 (18.5)	30 (16.6)	
50+	234 (81.5)	151(83.4)	
Grade: *N* (%)			**<0.001**
1,2	137 (50.2)	34 (20.7)	
3	136 (49.8)	130 (79.3)	
NA	14	17	
TNM Stage: *N* (%)			0.623
0	14 (4.9)	15 (8.2)	
1	55 (21.8)	40(26.3)	
2	103(40.9)	53 (34.9)	
3	68 (27)	43 (28.3)	
4	26 (10.3)	16 (10.5)	
NA	21	14	
Surgery: *N* (%)			0.062
Mastectomy	123 (50.8)	85(60.7)	
Wide local excision	119 (49.2)	55(39.3)	
NA	45	41	
Radiotherapy: *N* (%)			0.338
No	56 (22.1)	41 (26.3)	
Yes	197 (77.9)	115 (73.7)	
NA	34	25	
Adjuvant chemotherapy: *N* (%)			0.891
No	97 (36.2)	59 (35.5)	
Yes	171 (63.8)	107 (64.5)	
NA	19	15	
Neo-adjuvant chemotherapy: *N* (%)			0.387
No	195 (78.3)	105 (74.5)	
Yes	54 (21.7)	36 (25.5)	
NA	38	40	
Trastuzumab: *N* (%)			0.844
No	100 (35.2)	65 (36.1)	
Yes	184 (64.8)	115 (3.9)	
NA	3	1	
Neo-adjuvant Trastuzumab	38 (13.2)	32 (17.7)	0.261
pCR	10 (26.3)	13 (40.6)	0.368
Total	52 (18.1)	42 (23.2)	0.181
LRR	18 (6.3)	22 (12.2)	**0.027**
Distant	47 (16.4)	32 (17.7)	0.714

Bold values indicate significant *p* value

Overall, 299 (63.9%) patients were treated with Trastuzumab (Trast +ve). The clinical pathological details of the cohort are listed in Table 1, demonstrating the two subtypes are relatively matched for age, stage and treatment. The only statistical significant difference between the Luminal B HER2 and HER2+(ER−) was observed in the grade category, where the HER2+(ER−) cohort had a higher proportion of grade 3 cancers (49.8 vs. 79.3% *p* < 0.001). Furthermore, 263 (91.6%) of the Luminal B HER2 cancers received adjuvant hormone therapy. In the series recurrence occurred in 94 (20.1%) patients, of which 15 (3.2%) had LRR alone. 54 (11.5%) patients had distant metastasis alone and 25 (5.3%) patients had both LRR and distant metastasis. There was no significant difference in the distribution of age, stage or treatment of cancers between the two subtypes (Table 1).

### Trastuzumab treatment and breast cancer subtype significantly affects survival

#### Univariate analysis of survival

**Fig. 1:**
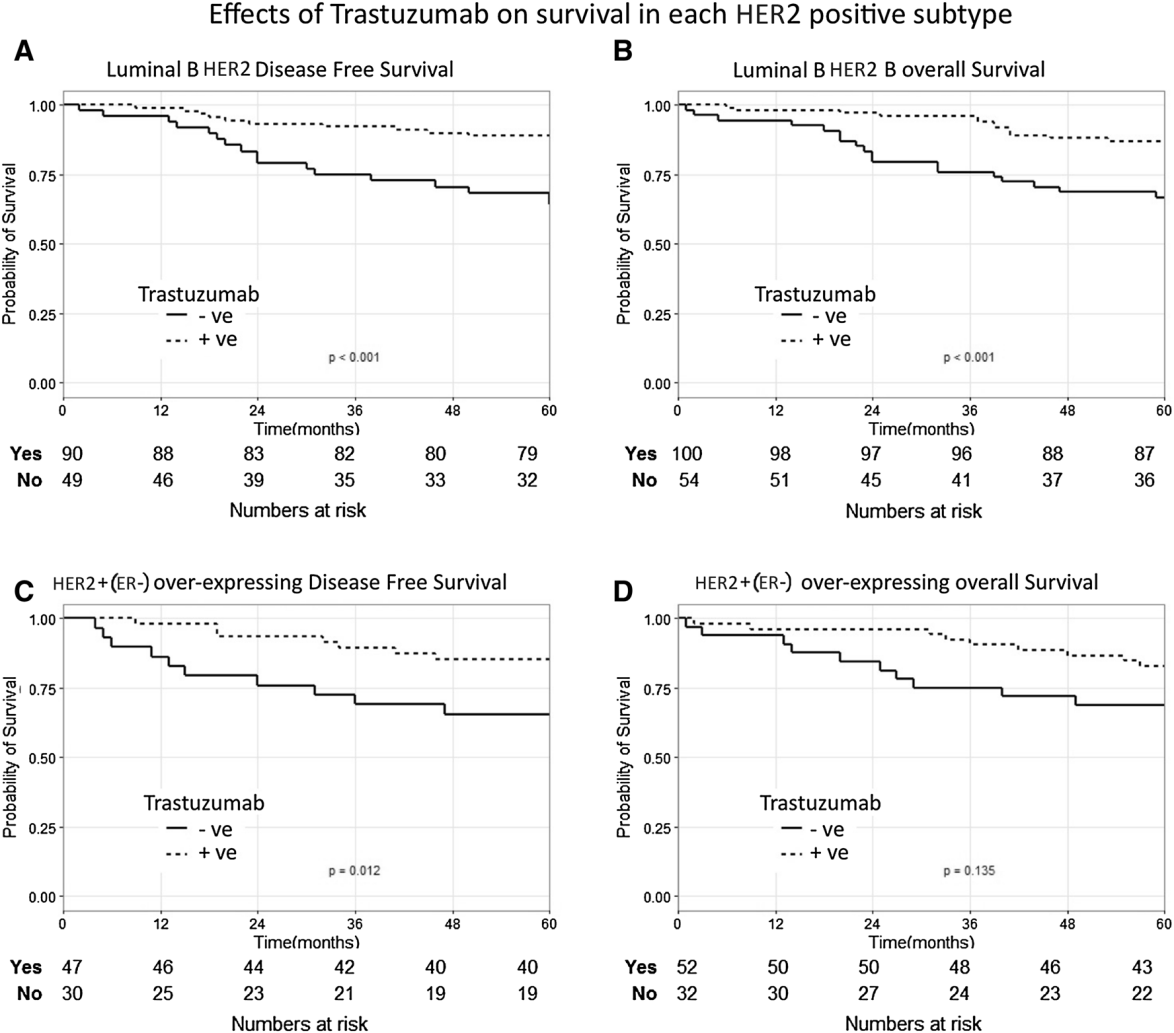
Kaplan–Meier curves of individual HER2 receptor-positive breast cancer subtypes. **a**, **b** Luminal B HER2 DFS and OS (respectively). **c**, **d** HER2+(ER−) DFS and OS (respectively). *DFS* Disease-Free Survival, *OS* Overall survival, Trast +ve: Patients treated with Trastuzumab, Trats −ve +: Patients who did not receive Trastuzumab

Survival was similar in Luminal B HER2 compared to HER2+(ER−) subtypes (Figure S1) and Trastuzumab treatment significantly improved overall survival in both subtypes (Figure S2). No difference was seen in survival between the two subtypes in either the Trast –ve or Trast +ve patients. Next to assess the impact of Trastuzumab introduction on each subtype, the 5-year DFS and OS were compared between the Trast –ve and Trast +ve groups in both subtypes. Analysing the DFS and OS by subtype, an increased survival rate is seen for Trast +ve patients in both Luminal B HER2 and HER2+(ER−) patients. However, a greater improvement was seen in Luminal B HER2 patients. Luminal B HER2 cancers had a statistically significant improvement in both 5-year DFS (*p* < 0.001) and OS (*p* < 0.001) (Fig. 1a, b), while the HER2+(ER−) only had a significant improvement in DFS (*p* = 0.012) but not OS (*p* = 0.135) (Fig. 1c, d).

### Multivariate analysis of survival

**Fig. 2:**
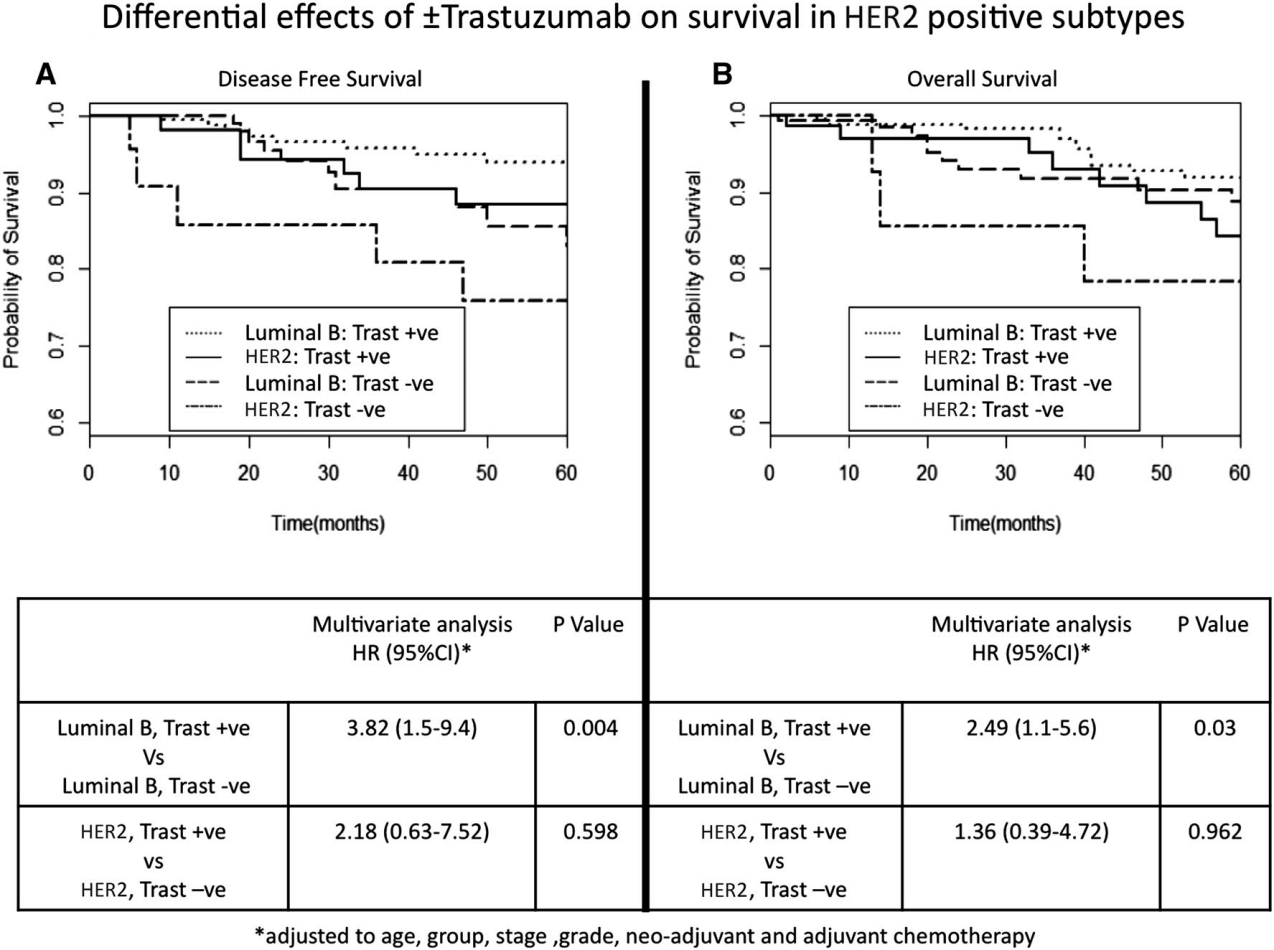
Cox proportional analysis of DFS and OS for patients treated with Trastuzumab. **a** Comparing DFS of Luminal B HER2 to HER2+(ER−) subtypes, Yes and No Trastuzumab treatment. **b** Comparing OS of Luminal B HER2 to HER2+(ER−) subtypes, Yes and No Trastuzumab treatment. *DFS* Disease-Free Survival, *OS* Overall survival, Trast +ve: Patients treated with Trastuzumab, Trats −ve +: Patients who did not receive Trastuzumab

No significant increased risk was seen in the HER2+(ER−) subtype when compared to Luminal B HER2 cancers for either 5-year DFS (HR 1.31, 95% CI 0.42–4.1) or 5-year OS (HR 2.18, 95% CI 0.79–6.03) (Table S1). Analysing risk factors for survival, higher grade was not associated with worse outcome but Trastuzumab given in the neo-adjuvant setting was associated with a significant improvement in 5-year DFS (HR 0.16, 95% CI 0.04–0.63). A multivariate cox proportional hazard model analysis of survival was performed, where the model was controlled for age, stage, grade and chemotherapy treatment (Fig. 2). A similar outcome is seen to the univariate analysis, with a greater improvement in survival seen in Luminal B HER2 patients treated with Trastuzumab. In Luminal B HER2 cancers, a significantly increased hazard ratio is seen in Trast –ve patients: DFS (HR 3.82, 95% CI 1.5–9.4; *p* = 0.004) and OS (HR 2.49, 95% CI 1.1–5.6; *p* = 0.03). However, no significant increase in hazard ratio was seen in Trast –ve HER2+(ER−) cancers compared to the Trast +ve group in 5-year DFS (HR 2000.18, 95% CI 0.63–7.52; *p* = 0.598) or OS (HR 1.36, 95% CI 0.39–4.72 *p* = 0.962).

### Effects of Trastuzumab treatment on recurrence rates

#### Univariate analysis of recurrence

Recurrences occurred in 52 (18.1%) of Luminal B HER2 breast cancers and 42 (23.2%) of HER2+(ER−) breast cancers overall. A significant reduction in recurrence rates in Trast +ve patients was observed in both Luminal B HER2 (38.3 vs. 8.5%, *p* < 0.001) and in HER2+(ER−) (36.7 vs. 18.3%, *p* = 0.009) (Table 2). Luminal B HER2 cancers displayed a significant reduction in LRR (16 vs. 1.8%, *p* < 0.001); however, there was no significant reduction in HER2+(ER−) breast cancers (16.7 vs. 10.6%, *p* = 0.261). Trastuzumab treatment induced a significant reduction in distant metastasis rates in both subtypes, with a greater reduction observed in Luminal B HER2 (36.2 vs. 6.7%, *p* < 0.001) compared to HER2+(ER−) (31.7 vs. 12.5%, *p* = 0.03).

**Table 2:** Recurrence rates (stage I–III breast cancers)

	Trastuzumab	Luminal B (*n* = 259) *N* (%)	HER2 (*n* = 164) *N* (%)
Total Recurrence: *n* (%)	No	36/94 (38.3)	22/60 (36.7)
	Yes	14/165 (8.5)	19/104 (18.3)
		***p*** **<** **0.001**	***p*** **=** **0.009**
LRR: *n* (%)	No	15/94 (16)	10/60 (16.7)
	Yes	3/165 (1.8)	11/104 (10.6)
		***p*** **<** **0.001**	*p* = 0.261
Distant: *n* (%)	No	34/94(36.2)	19/60 (31.7)
	Yes	11/165(6.7)	13/104 (12.5)
		***p*** **<** **0.001**	***p*** **=** **0.003**

Bold values indicate significant *p* value

*LRR* Local Regional Recurrence, *Distant* distant metastasis

Analysis of the effects of Trastuzumab treatment on distant metastasis by site of recurrence and subtype was performed (Table 3). For Trast –ve patients, bone was the most common site of metastasis for Luminal B HER2 cancers, while lung was the most common site in HER2+(ER−). Following Trastuzumab treatment in the Luminal B HER2 cancers, a significant reduction was seen for all distant sites of metastasis (except brain). The site with the greatest reduction in metastasis, due to Trastuzumab treatment, was in bone (22.9 vs. 3.8%, *p* < 0.001). HER2+(ER−) breast cancers did show decreases in all metastatic sites except for brain in Trast +ve patients; however, these reductions only approached significance in bone (*p* = 0.075), lung (*p* = 0.086) and liver (*p* = 0.075).

**Table 3:** Distant metastasis rates

Metastasis site	Luminal B		*p* Value	HER2		*p* Value
	No Trastuzumab *N* = 86	Trastuzumab *N* = 173		No Trastuzumab *N* = 60	Trastuzumab *N* = 104	
Bone: *n* (%)	24 (25.5)	5 (3)	**<0.001**	6 (10)	3 (2.1)	0.075
Brain: *n* (%)	4 (4.3)	4 (2.4)	0.466	5 (8.3)	9(8.7)	0.999
Lung: *n* (%)	20 (21.3)	3 (1.8)	**<0.001**	9 (15)	7 (6.7)	0.086
Liver: *n* (%)	13 (13.8)	7 (4.2)	**0.005**	6 (10)	3 (2.9)	0.075

Bold values indicate significant *p* value

### Multivariate analysis of recurrence

Performing a multivariate analysis of recurrence risk by treatment, metastatic site and subtype (Table 4), no difference was seen for LRR between Luminal B HER2 and HER2+(ER−) cancer in Trast –ve patients (OR 1.39, 95% CI 0.47–4.5: *p* = 0.557). Importantly in Trast +ve patients, a significantly lower odds ratio for LRR was seen in Luminal B HER2 cancers compared to HER2+(ER−) (OR 0.13, 95% CI 0.02–0.59; *p* = 0.018). Distant metastasis rates to bone were the only significant difference between the two subtypes, in the no Trastuzumab group (OR 4.63, 95% CI 1.53–17.5; *p* = 0.012). Following Trastuzumab treatment, no difference was seen between subtypes in bone metastasis (OR 0.958, 95% CI 0.17–5.60; *p* = 0.96) but a significantly lower risk in brain metastasis is seen in the Luminal B HER2 cancers (OR 0.19, 95% CI 0.03–0.85; *p* = 0.041).

**Table 4:** Logistic regression analysis (site of recurrence)

	Unadjusted OR# No Trastuzumab	*p* Value	Adjusted OR# No Trastuzumab*	*p* Value	Unadjusted OR# Trastuzumab	*p* Value	Adjusted OR# Trastuzumab*	*p* Value
	Luminal B versus HER2		Luminal B versus HER2		Luminal B versus HER2		Luminal B versus HER2	
LRR	0.95 (0.399,2.338)	0.907	1.39 (0.471,4.50)	0.557	0.16 (0.04,0.51)	**<0.001**	0.13 (0.02,0.59)	**0.018**
Distant	1.22 (0.618,2.459)	0.566	1.21 (0.508,2.95)	0.660	0.50 (0.211,1.16)	0.107	0.51 (0.17,1.44)	0.214
Bone	3.09 (1.246,8.805)	**0.022**	4.63 (1.53,17.50)	**0.012**	1.05 (0.253,5.22)	0.945	0.958 (0.17,5.60)	0.960
Brain	0.49 (0.117,1.923)	0.301	0.41 (0.09,1.76)	0.231	0.26 (0.07,0.829)	**0.029**	0.19 (0.03,0.85)	**0.041**
Lung	1.53 (0.661,3.786)	0.333	2.15 (0.78,6.688)	0.161	0.26 (0.05,0.946)	0.05	0.36 (0.06,1.57)	0.198
Liver	1.44 (0.536,4.324)	0.483	1.03 (0.33,3.39)	0.958	1.49 (0.405,7.04)	0.569	1.03 (0.22,5.64)	0.971

Bold values indicate significant *p* value

# 95% CI

* Adjusted to stage and grade

*LRR* Local Regional Recurrence, *DFS* Disease-Free Survival, *OS* Overall survival

## Discussion

The impact of Trastuzumab treatment has been well established, greatly improving survival and significantly reducing recurrence in HER2 receptor-positive breast cancers [15, 21, 22]. It has also been shown to improve pathological complete response rates, and in our study pCR was associated with an improved DFS. A pooled analysis of 11,955 patients in neo-adjuvant chemotherapy trials found that, overall, patients achieving pCR had a higher level DFS than those with residual cancer [23]. However, few studies have investigated the differential effects of Trastuzumab treatment on Luminal B HER2 and HER2+(ER−) breast cancers. Our analysis revealed that in the Trastuzumab era, Luminal B HER2 cancers specifically had a greater improvement in overall survival. Interestingly, while Trastuzumab treatment resulted in a significant reduction in LRR rates for Luminal B HER2 cancers, only a modest improvement was seen for the HER2+(ER−) subtype. In both subtypes, Trastuzumab treatment resulted in significant reductions in overall rates of distant metastasis at all sites, except for brain metastases. Furthermore, a greater reduction was seen in Luminal B HER2 breast cancers, with the largest reduction in overall metastasis rates seen in the bone metastasis.

Few previous studies have compared survival or recurrence rates between the two HER2 receptor-positive breast cancers since the introduction of Trastuzumab. Romond et al. compared survival between the two HER2 receptor-positive breast cancers and found at 4-year follow-up that hormone receptor status minimally influenced the response to Trastuzumab, although hormone receptor status was reported as a significant predictor in DFS [15]. Our findings support previous findings where Trastuzumab treatment led to a reduction in LRR only in Luminal B HER2 cancers [24]. We demonstrate a statically significant reduction in LRR rates observed in Luminal B HER2 patients, while no significant difference was seen in the HER2+(ER−) subtype.

Previous studies demonstrated that bone was the most common recurrence site in Luminal B HER2 breast cancers and lung was most common for HER2+(ER−) [8, 9]. We show Trastuzumab treatment resulted in a reduction in metastasis to all sites, except the brain which is explained by the fact that Trastuzumab does not cross the blood/brain barrier [25, 26]. Surprisingly, Luminal B HER2 cancers treated with Trastuzumab showed the greatest reduction in distant metastasis to the bone. While Trastuzumab treatment did not lead to significant variations in brain metastasis rates, it did result in the Luminal B HER2 cancers having a significantly reduced odds ratio of brain metastasis. This correlates with previous studies which show a higher brain metastasis rates in hormone receptor-negative tumours [26].

A potential reason for variation between subtypes could be that HER2 receptor over expression reduces the response to hormone therapy. Studies have observed increased hormone resistance rates in Luminal B HER2 cancers compared to Luminal A cancers [27, 28]. Cross talk between HER2 receptors and hormone receptors results in activation of the hormone receptor, even in the presence of hormone treatment [29]. Clinical trials have shown that the addition of Trastuzumab to hormone treatment improves survival in metastatic breast cancer [30]. In our study, the introduction of Trastuzumab significantly reduced distant metastasis, especially in bone metastasis rates. By reducing the activity of HER2 receptors, Trastuzumab may restore the response to hormone therapy in Luminal B HER2 cancers [31]. In the neo-adjuvant setting, this may provide an explanation as to why lower levels of pCR are seen in Luminal B HER2 cancers compared to HER2+(ER−) cancers [23].

Another potential difference between subtypes could be the increased number of grade 3 cancers seen in the HER2+(ER−) group. It has been shown that both HER2+(ER−) and triple-negative breast cancers have worse outcomes and present with higher-grade cancer than Luminal cancers [6]. Although, in a pooled analysis of neo-adjuvant Trastuzumab trials, grade 3 cancers had a higher pCR rate than grade 1 and 2 cancers [23]. Importantly, our study revealed that on multivariate analysis of patients treated with Trastuzumab, grade 3 cancers did not have a lower DFS or OS risk when compared to grade 1 and 2 breast cancers.

In patients receiving neo-adjuvant Trastuzumab chemotherapy, around 40% of patients will have a complete pathological response [20, 32]. This shows that a large proportion of patients only have a partial or no response to Trastuzumab treatment. This resulted in the development of new anti-HER2 receptor treatments targeting different pathways such as Pertuzumab, which has been shown to increase response [33]. Our study highlights the improved prognosis associated with anti-HER2 receptor therapy; it also demonstrates that a large proportion of patients survived despite not being treated with Trastuzumab. We believe this clearly indicates that Trastuzumab treatment is not required for all HER2 receptor-positive breast cancers. There is a clear need to develop a molecular or genetic scoring system to identify which patients will benefit from Trastuzumab treatment and those that will not.

This study once again shows the benefit of Trastuzumab treatment in HER2 receptor-positive breast cancers, demonstrating the effects on both survival and recurrence rates. It also highlights how a targeted therapy has altered responses in related breast cancer subtypes, emphasising their molecular differences. Demonstrating a more positive impact of Trastuzumab treatment on Luminal B HER2 cancers supports the need to further characterise the mechanism of action of Trastuzumab in each subtype, suggesting that key differences remain to be defined. This work highlights the need to fully understand the subtype-specific effects and mechanisms of action of Trastuzumab therapy, which will allow truly individualised breast cancer management regimes to be implemented.

## Electronic supplementary material

Below is the link to the electronic supplementary material.

Supplementary material 1 (DOCX 267 kb)
