# Supplementary material for: Differential impact of hormone receptor status on survival and recurrence for HER2 receptor-positive breast cancers treated with Trastuzumab
Source: Breast Cancer Res Treat. 2017 Apr 4;164(1):221–9. doi: 10.1007/s10549-017-4225-5 (PMC5487720; doi:10.1007/s10549-017-4225-5)
Supplement: Supplementary file 1 — Supplementary material 1 (DOCX 267 kb) [file 10549_2017_4225_MOESM1_ESM.docx]

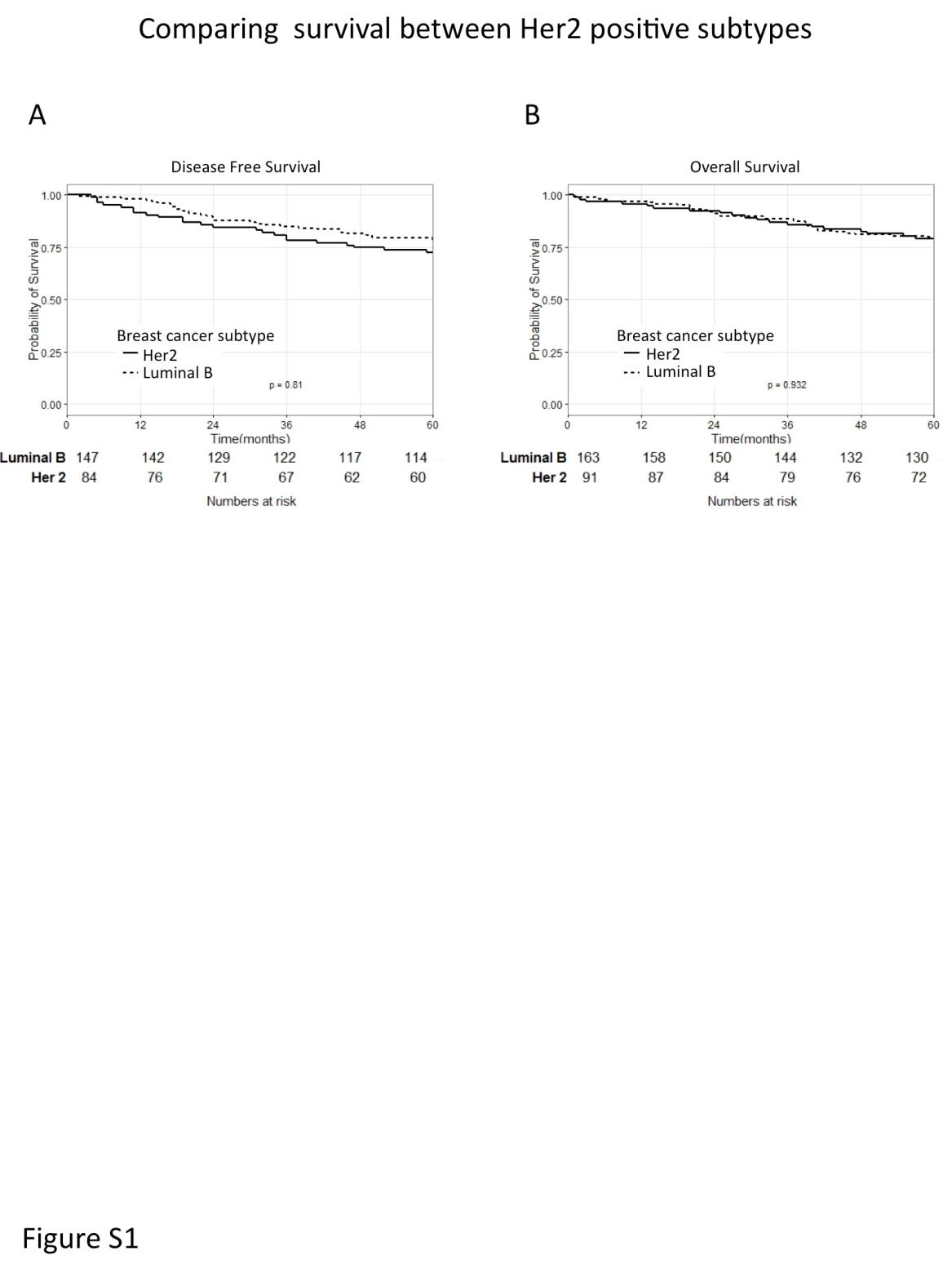

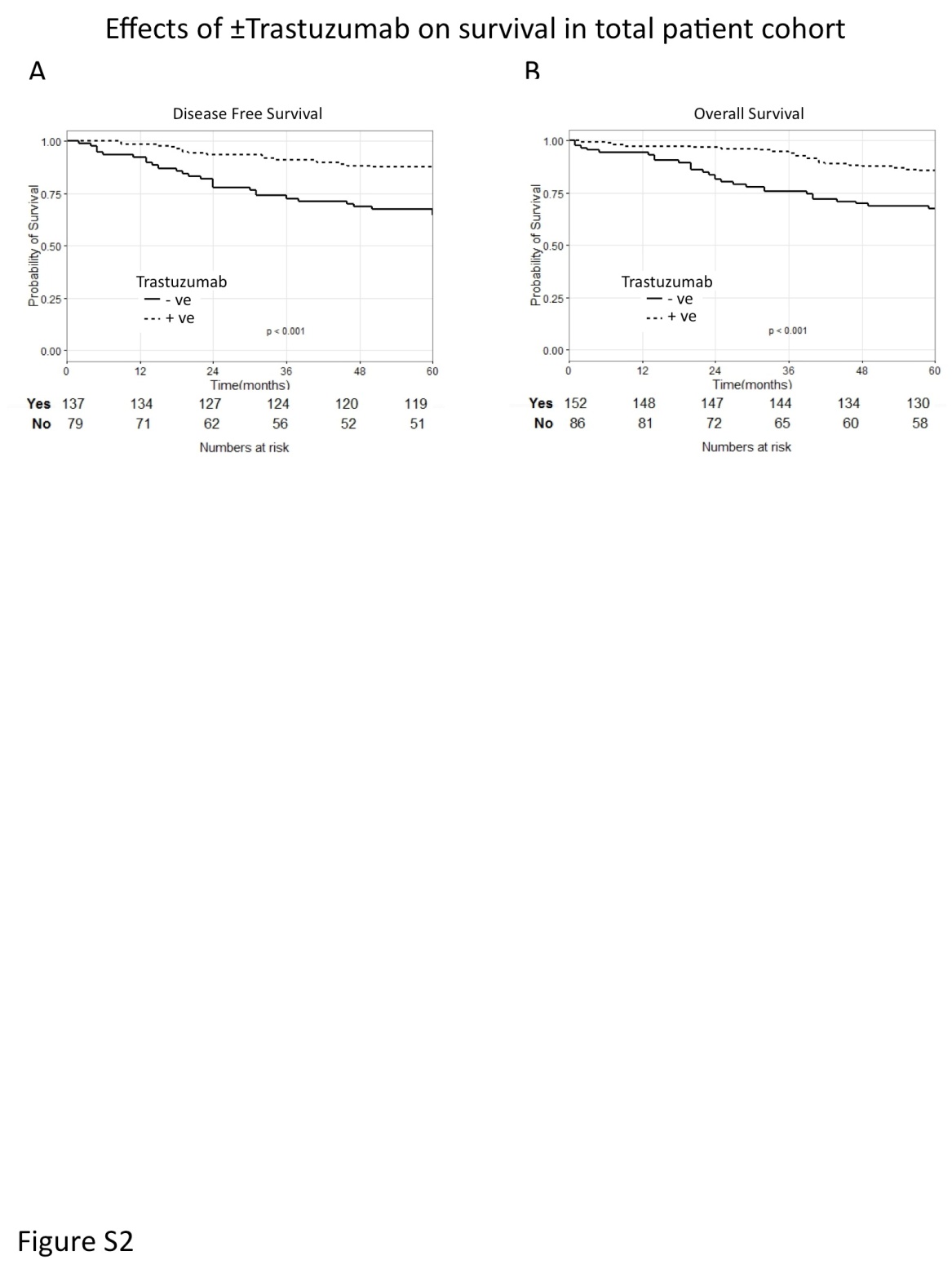


| Table Supplemental 1. Multivariate analysis of patients treated with Trastuzumab | | |
| --- | --- | --- |
|  | **DFS** | **OS** |
|  | Multivariable HR(95%CI) | Multivariable HR(95%CI) |
| **Subtype** | | |
| Luminal B | 1 | 1 |
| Her 2 | 1.31 (0.42 – 4.1) | 2.18 (0.79 – 6.03) |
| **Age Category** | | |
| 0 to 50 | 1 | 1 |
| 50+ | 1.16 (0.31 – 4.29) | 1.16 (0.3 – 4.46) |
| **Grade** | | |
| 1,2 | 1 | 1 |
| 3 | 1.16 (0.34 – 3.93) | 0.56 (0.21 – 1.49) |
| **TNM Stage** | | |
| 1 | 1 | 1 |
| 2 | 2.29 (0.25 – 21.01) | 0.95 (0.09 -10.54) |
| 3 | 12.64* (1.51 – 105.62) | 7.48 (0.92 – 61.03) |
| 4 |  | 40.68* (5.21 -317.88) |
| **Adjuvant Chemotherapy** | | |
| No | 1 | 1 |
| Yes | 0.42 (0.09 – 2.0) | 0.40 (0.07 – 2.37) |
| **Neo-adjuvant Chemotherapy** | | |
| No | 1 | 1 |
| Yes | 0.16* (0.04 – 0.63) | 0.44 (0.12 – 1.53) |
| * p<0.05 |  |  |

DFS: Disease Free Survival; OS: Overall survival.
